# How diet, physical activity and psychosocial well-being interact in women with gestational diabetes mellitus: an integrative review

**DOI:** 10.1186/s12884-019-2185-y

**Published:** 2019-02-07

**Authors:** Leah Gilbert, Justine Gross, Stefano Lanzi, Dan Yedu Quansah, Jardena Puder, Antje Horsch

**Affiliations:** 1 Obstetric service, Department Woman-Mother-Child, Lausanne University Hospital, 1011 Lausanne, Switzerland; 2 Service of Endocrinology, Diabetes and Metabolism, Lausanne University Hospital, 1011 Lausanne, Switzerland; 3 Division of Angiology, Heart and Vessel Department, Lausanne University Hospital, 1011 Lausanne, Switzerland; 4 Institute of Higher Education and Research in Healthcare (IUFRS), University of Lausanne, 1010 Lausanne, Switzerland; 5 Neonatology service, Department Woman-Mother-Child, Lausanne University Hospital, 1011 Lausanne, Switzerland

**Keywords:** Intervention, Exercise, Nutrition, Mental health, Pregnancy

## Abstract

**Background:**

Gestational Diabetes Mellitus (GDM) is associated with future cardio-metabolic risks for the mother and her child. In addition, one-third of women with recent GDM develop postpartum depression. Given these adverse impacts of GDM on the health of the mother and her offspring, it is important to intervene on modifiable factors, such as diet, physical activity, and psychosocial well-being. This integrative review therefore explored evidence on how these modifiable factors interact in women with GDM and their offspring, and how effective combined interventions are on reducing adverse impacts of GDM.

**Methods:**

A comprehensive search strategy included carefully selected terms that corresponded to the domains of interest (diet, physical activity and psychosocial well-being). The databases searched for articles published between 1980 and February 2018 were: CINAHL, PsycINFO, Embase, Pubmed and Cochrane. Studies that were included in this review were either observational or intervention studies that included at least two domains of interest. Articles had to at least report data on maternal outcomes of women with GDM.

**Results:**

The search strategies identified 14′419 citations after excluding duplicates. After screening titles and then abstracts, 114 articles were selected for detailed evaluation of their full text, and 16 were included in this review: two observational and 14 intervention studies. Results from observational studies showed that psychosocial well-being (social support and self-efficacy) were positively associated with physical activity and dietary choice. Intervention studies always included diet and physical activity interventions, although none integrated psychosocial well-being in the intervention. These lifestyle interventions mostly led to increased physical activity, improved diet and lower stress perception. Many of these lifestyle interventions also reduced BMI and postpartum diabetes status, improved metabolic outcomes and reduced the risk of preterm deliveries and low birth weight.

**Conclusion:**

This integrative review showed that psychosocial well-being interacted with diet as well as with physical activity in women with GDM. We recommend that future studies consider integrating psychosocial well-being in their intervention, as observational studies demonstrated that social support and self-efficacy helped with adopting a healthy lifestyle following GDM diagnosis.

**Electronic supplementary material:**

The online version of this article (10.1186/s12884-019-2185-y) contains supplementary material, which is available to authorized users.

## Introduction

Gestational Diabetes Mellitus (GDM) is defined when a women has a glucose intolerance with onset and first recognition between 24 to 28 weeks of gestation [1, 2]. It usually resolves after childbirth [2, 3], although it carries pre-, peri-, and postnatal risks of adverse outcomes in the mother and the child [1]. For example, up to 40% of women with GDM are known to have pre-diabetes in the early postpartum period [4]. The prevalence of GDM is 10.8% in Switzerland [5], 9.2% in the USA [6], 6.8% in China [7], 16.3% in Qatar [8] and 7.8% among a racially/ethnically diverse population [9].

Mothers have a risk of up to 70% of GDM recurrence, a seven-fold higher five to 10 year risk of type 2 diabetes, and an increased risk of cardiovascular diseases [10–13]. GDM is also associated with reduced psychosocial well-being: women with GDM are two to four times [14] more likely to develop antenatal or postpartum depression. Evidence shows that approximately one-third of women with recent GDM develop postpartum depression [15]. Postpartum depression in turn is associated with an increase in comfort eating and a decrease in physical activity [16], thus putting the women at higher risk of weight gain and future diabetes [15].

With regards to negative consequences for the child, GDM is associated with macrosomia at birth (> 4 kg birth weight), excess body fat and paediatric obesity [17–24]. Intrauterine exposure to GDM also doubles the risk for type 2 diabetes in the children of GDM mothers [25]. Apart from GDM, maternal pre-pregnancy overweight and excessive gestational weight gain also predict higher birth weight and adiposity during infancy [26, 27]*.* Furthermore, maternal lifestyle behaviour, such as a high fat diet or lack of physical activity during pregnancy, can influence offspring adiposity independent of maternal obesity [27, 28].

Given the deleterious impact of GDM during pregnancy on the health of the mother and her offspring, it appears crucial to work on modifiable risk factors during the pre-, peri-and postnatal period, namely diet, physical activity, and psychosocial well-being [29]. Excessive gestational weight gain [30] is very frequent in women with GDM and strongly associated with lifestyle factors during pregnancy [31]. High fat consumption particularly saturated fat, trans fat and cholesterol, increases GDM risk [32–34]. A higher intake of added sugar and lower intake of vegetable and fruit fiber are independently linked to increased fasting glucose [34]. Animal protein intake is positively and vegetable protein inversely associated with GDM risk [35]. Another important domain that can address risk factors of GDM is physical activity, which decreases insulin resistance, reduces future risk of type 2 diabetes [36], and limits gestational weight gain by increasing energy expenditure and altering food intake [37]. Thus, physical activity has a protective effect on the development of GDM [38, 39]. Finally, psychological factors also play an important role in GDM. Higher stress exposure and perceived stress are associated with increased fasting glucose levels in pregnant women, even before they know their diagnosis [40]. Psychological stress and negative life events can be associated with higher salivary cortisol levels during pregnancy, which might influence glucose levels [41]. Depressive symptoms in early pregnancy also increase the risk for GDM [14, 39].

Many modifiable risk factors that relate to GDM also interact with each other. In this review, the term “interaction” covers correlations or associations, found in the original papers, between our domains of interest [diet (including breastfeeding), physical activity and psychosocial well-being (including depression, anxiety, stress, sleep, self-efficacy and social support)]. For example, physical activity may reduce symptoms of depression [42], probably by reducing plasma kynurenine [43, 44]. Physical activity increases energy expenditure [45], can influence total food intake [45, 46], reduces stress-induced food intake [47] and can also regulate eating behavior via endocrine mediators such as insulin, leptin, and ghrelin [48–50]. Eating behavior, such as emotional eating or unhealthy habitual eating plays an important role in explaining the depression-BMI relationship [51–55]. Finally, the higher risk for maternal postpartum depression is also associated with reduced parenting skills, which may have negative consequences for the development of the child [56–58]. Given the interaction of these domains, designing interventions that integrate more than one domain of interest (diet, physical activity and psychosocial well-being) may be promising. Many interventions in women with GDM focus on either diet [59–61], physical activity [62–66], or combined diet and physical activity interventions [67, 68]. However, to our knowledge, there are no interventions combining diet and/or physical activity with psychosocial well-being. Therefore, this integrative review explored how physical activity, diet, and psychosocial well-being interact in women with GDM and in their offspring by analyzing and synthetizing observational and intervention studies. In addition, we investigated how effective interventions that address more than one domain of interest are in reducing risk factors associated with GDM. Addressing these questions may help to identify effective ingredients of interventions to counter the negative impact of GDM in women and their offspring.

## Methods

### Design

This integrative review follows the guidelines elaborated by Whitemore and Knafl (2005) [69]. As we were investigating a new topic, we needed a design that would allow us to explore this topic in a broad manner and to produce evidence-based results. We followed Whitemore and Knafl’s design firstly by identifying variables of interest and elaborating specific research questions. We then used computerized databases to augment efficiency as well as the scope of our review. Secondly, we defined inclusion and exclusion criteria that guided the decision to exclude irrelevant articles, and we evaluated the quality of each original article. When analysing data, we categorized, summarized and ordered our data extracted from primary articles and organized the results according to subgroups. Whitemore and Knafl (2005) [69] also recommend creating data displays; thus, we summarized our findings in tables (see Additional file 1) and created a conceptual model integrating all of our results (see Fig. 2). Finally, we specified the implications for clinical practice, as recommended by the authors.

### Search strategy

**Fig. 1:**
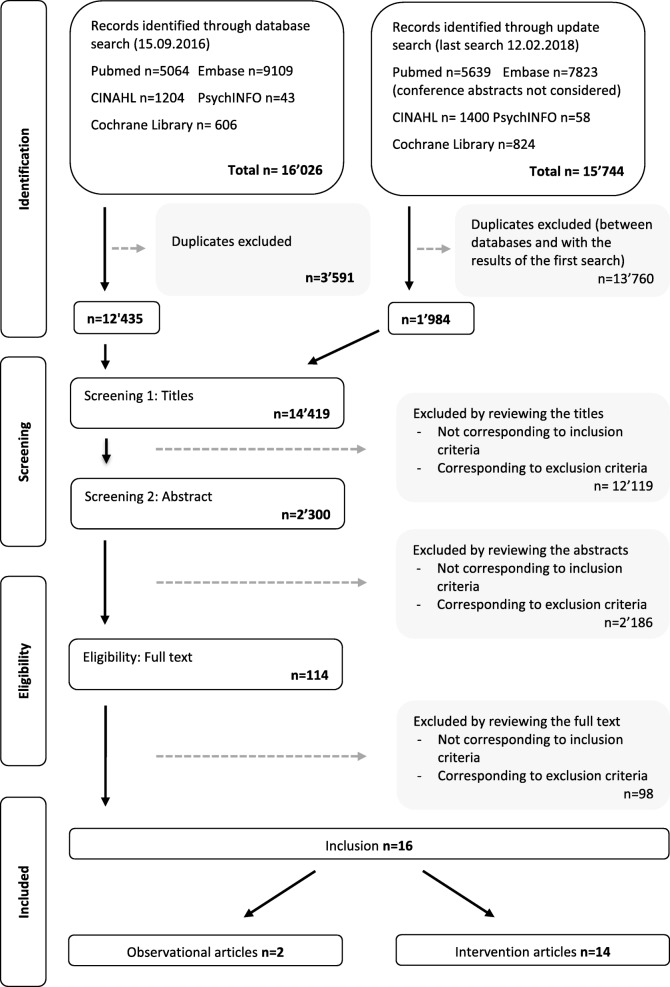
Prisma Flowchart. This Prisma flowchart illustrates the process through which articles for this integrative review were included or excluded

A comprehensive search strategy included carefully selecting terms that corresponded to the domains of interest [diet (including breastfeeding), physical activity and psychosocial well-being (including depression, anxiety, stress, sleep, self-efficacy and social support)] (please refer to Additional file 2 for details on the search strategy) by consulting a team of interdisciplinary experts and a specialised librarian. The databases searched for articles were: CINAHL, PsycINFO, Embase, for which usual subject headings were used and Pubmed, for which the strategy was completed with free-text terms to also collect the non-indexed articles, and finally, Cochrane, for which the strategy used only free search terms. All studies identified during the search were assessed for relevance to the review based on the information provided in the title and abstract. For all papers that appeared to meet the inclusion criteria, full papers were retrieved. Full papers were again assessed for eligibility in order to determine relevance to the review objective. The period considered was from 1980 to the date of the first search (September, 15, 2016) and this first search identified 16′026 articles. An update of the search was performed between the 15 of September 2016 and the 12 of February 2018 and identified 15′744 articles. This contained articles found in the first search as well as new ones; for this reason, a large number of duplicates were removed after the second search (13′760) (Fig. 1). The second literature search yielded fewer articles than the first one because we were able to exclude the time period related to our first search in Pubmed, thus avoiding the exclusion of duplicates in this database.

### Inclusion criteria

Inclusion criteria were either observational or intervention studies in women with GDM that focused on at least two domains of interest. Articles were published in English in peer-reviewed journals and had to contain data on women with GDM (or women and their partner), or previous GDM, with clinical outcomes reported for women (or women and their baby). The decision to include articles from 1980 was made in order to stay in line with more up-to-date clinical practice and objectives for glycemic thresholds.

### Exclusion criteria

We excluded study protocols, conference abstracts, recommendation papers, guidelines, qualitative studies, and review articles. Articles that exclusively investigated women with type 1 and type 2 diabetes were excluded. Intervention studies that only tested pharmacological interventions were also excluded, as were genetic, epigenetic and genomic studies. Studies on diet, which focused only on dietary supplements were also excluded. Animal research and papers addressing exclusively the microbiome were also excluded.

### Data extraction and quality appraisal

All identified citations were collated in a citation management system (Endnote X7) and duplicates were removed. The search strategies identified 14′419 citations after excluding duplicates (see above and Fig. 1). After screening titles and then abstracts, 114 articles were selected for detailed evaluation of their full text, and 16 were included in this review.

Data from the 114 articles were extracted systematically from all eligible papers with a modified Joanna Briggs Institute (JBI) data extraction form for review and research synthesis designed by LG. This allowed for sequential extraction of articles by LG and DYQ to make final decisions on which papers to include and those to exclude. Any disparities or disagreements were resolved by consensus-based discussions with AH.

Following this, JG and SL independently extracted the data and produced Additional file 1: Tables S1 and S2. The quality of included studies was assessed with the JBI critical appraisal *Checklist for Randomized Controlled Trials* [70], *Checklist for quasi-experimental studies (non-randomized experimental studies)* [71]*, Checklist for Analytical Cross Sectional Studies* [72] and *Checklist for Cohort studies* [73]. Two reviewers (LG & DYQ) undertook the quality assessment independently and later resolved discrepancies in score ratings by consensus. The appraisal checklists assessed the aims of the study, sampling procedure, data collection methods, main findings, and limitations.

### Synthesis of findings

Findings from the included studies were synthesized according to the objectives of the study in a thematic manner, as suggested by Whittemore and Knafl (2005) [69]. Firstly, links between the different domains of interest in the observational and intervention studies were synthesized, forming the base for a conceptual framework. Secondly, the effects of interventions on clinical outcomes were summarized.

## Results

### Characteristics of included studies

This review included 16 studies (Fig. 1): two observational studies and 14 intervention studies. The observational studies were conducted in the USA (*n* = 1/2) [74] and Switzerland (*n* = 1/2) [75], employing a cross-sectional (*n* = 1/2) [74] or a prospective cohort design (*n* = 1/2) [75]. The 14 intervention studies took place in eight different countries, with the highest number of them conducted in the USA (*n* = 5/14) [76–80] and China (*n* = 3/14) [81–83]. The remaining studies were carried out in Australia (*n* = 1/14) [84], Canada (*n* = 1/14) [85], Finland (*n* = 1/14) [86], Ireland (*n* = 1/14) [87], Spain (*n* = 1/14) [88] and Thailand (*n* = 1/14) [89]. Of these intervention studies, the large majority were randomized controlled trials (RCTs) (*n* = 9/14) [76, 78, 79, 81, 82, 84, 87–89] and the remaining studies were intervention trials (*n* = 5/14), with two of them (*n* = 2/5) containing a control group [80, 83] and the other three (*n* = 3/5) using a pre/post-test design [77, 85, 86]. The majority of the included studies were published between 2011 and 2015 (*n* = 10/16) [76, 77, 81, 83–89], whereas the remaining studies were published between 2006 and 2010 (*n* = 3/16) [74, 78, 80] and 2016–2018 (*n* = 2/16) [75, 82]. One study (*n* = 1/16) [79] was published in 1989.

All combined intervention studies focused solely on diet and physical activity and none included psychosocial well-being in their intervention. For reasons of simplicity and clarity, these combined studies will be named “lifestyle interventions”.

In these intervention studies, the extracted data for this review focused on outcomes of the intervention groups which were always compared to the respective other GDM control groups; thus we will not mention this in our result section, to increase readability. Only one intervention study (*n* = 1/14) [83] contained more than two groups. Indeed, this study had five different groups (lifestyle, diet only, physical activity only, no intervention and a “no GDM” group). We chose to report results for the lifestyle intervention compared to the “no intervention group” only, to be in line with the other studies integrated in this review. An exception remains for three studies (*n* = 3/14) that were designed differently. Indeed, one study compared the lifestyle intervention group at the end of the study (1 year) to the baseline of that same lifestyle intervention group and thus did not contain a control group (*n* = 1/14) [86]. For the second study, the authors used a single-group pre-post design and measured the effect of the intervention across time [77]. Finally, the last study was a single arm pilot before and after intervention study [85]. For these studies, these design details will always be mentioned in our results section.

Lifestyle interventions lasted from 6 weeks [79] to 4 years [82] and either contained results at the end of the intervention [76, 78–86, 88, 89] or, for only two studies, after a follow-up period [77, 87]. As time effects (baseline to end of the study or to follow-up) are always present in initial papers for intervention groups and given that they vary largely, they will be mentioned in detail in our results section.

### Study participants

A total of 20,285 participants were included in the studies, with *n* = 19,884 in the intervention and *n* = 401 in the observational studies. The lowest number of participants in a study was 17 [85] and the largest study consisted of 14,168 participants [83].

### Associations measured in the observational studies

Both observational studies investigated the associations between diet, physical activity and psychosocial variables (*n* = 2/2) [74, 75]. Specifically, the authors assessed the link between social support and diet and physical activity, and the relationship between psychosocial well-being (self-efficacy, social support and self-efficacy and social support) and diet and physical activity.

### Lifestyle interventions investigated

The lifestyle intervention adopted in all intervention studies consisted of combined diet and physical activity interventions (*n* = 14/14). The dietary components of the study interventions required participants to follow either the American Diabetes Association (ADA) diet [76], the Diabetes Prevention Program (DPP) guidelines [77, 78], the Canada’s Food Guide [85], a Mediterranean diet [88] or other types of dietary guidelines [79–84, 86, 87, 89, 90]. In most studies, participants were advised to either conduct moderate to vigorous physical activity for around 150 min a week [76–78, 85] or 30 min a day [81, 82], to be more active and incorporate light and moderate physical activity as much as possible in daily life [83], to increase the number of steps (walking) a day to 10′000 [84], or to have a specific yoga routine (nine postures) [89]. In four studies, participants were asked to exercise at moderate intensity [79, 80, 87, 88]. In one study, intervention participants were provided with a study pedometer to track their daily steps [84]. In another study [86], training with a coach provided empowerment during physical activity.

All outcome variables were tested either during pregnancy [80, 83, 89] or in the postpartum period [74–88] In intervention studies, the interventions started either during pregnancy [76, 79, 80, 82, 83, 89] or in the postpartum period [77, 78, 81, 84–88] (for details, please refer to Additional file 1: Tables S1 and S2).

### Interactions between domains of interest (diet, physical activity and psychosocial well-being)

Focusing on the *observational studies*, one prospective observational study (*n* = 1/2) [75] revealed that the main normative influences for healthy behaviors (diet and physical activity) were the husband/partner (68%) and other family members (56%). After controlling for significant individual factors, the study showed that a lower level of social support was related to a lower adherence to a healthy lifestyle in the postpartum period.

Regarding diet and its relationship with self-efficacy, the authors of the cross-sectional study [74] showed that women reported low self-efficacy for not overeating. They further demonstrated that self-efficacy for not overeating was associated with better dietary quality, although this association missed significance after adjusting for covariates.

In terms of the relationship between diet and social support, women reported moderate social support for consuming a healthy diet [74]. Higher social support from both friends and family for a healthy diet correlated with better dietary quality, with a trend towards statistical significance. The authors further demonstrated that after adjustment for covariates, stronger social support from family and friends for dietary habits was associated with better dietary quality.

Regarding physical activity and its relationship with self-efficacy, Kim et al. (2008) demonstrated in the cross-sectional observational study that women reported low self-efficacy for physical activity [74]. However, greater self-efficacy for physical activity was associated with a greater number of hours spent walking and greater leisure time spent in vigorous intensity activity, but not with walking intensity. When the authors adjusted their analysis for covariates, greater self-efficacy for physical activity was associated with more than 4 hours per week spent walking and with spending at least 20 min three times a week in a vigorous activity.

Regarding associations between physical activity and social support, the cross-sectional study (*n* = 1/2) [74] observed that women reported moderate social support for physical activity. Furthermore, they observed that social support from friends for physical activity was associated with a greater number of hours spent walking and greater leisure time spent in vigorous activity. Moreover, social support for physical activity was associated with greater leisure time physical activity, but not with the total number of hours spent walking. Furthermore, after adjustment for covariates, social support from friends was also associated with more than 4 hours spent walking per week, but not with walking intensity and leisure time activity. In the prospective observational study (*n* = 1/2) [75] observing the link between social support and physical activity, women indicated a need for personalized advice (65%) and sport facilities where their children can be looked after (69%) to facilitate their physical activity practice.

All *intervention studies* were combined physical activity and diet lifestyle interventions (*n* = 14/14). The lifestyle interventions led to a decreased fat intake in two studies, one during the intervention period (7 months) (*n* = 1/2) [76] and one at 6 months follow-up after a three-month intervention, compared to baseline [77] (*n* = 1/2). In one study, the lifestyle intervention lead to a higher diet adherence at the one-year follow-up after a 12-week intervention (*n* = 1/1) [87]. Higher diet self-efficacy was seen in two studies, once at one-year follow-up after a three-month intervention [87] and also at the end of a three month intervention [84]. In addition, there was a higher proportion of women who partially or exclusively breastfed during the intervention (7 months) in one study (*n* = 1/1) [76]. Other outcomes for diet in the lifestyle intervention studies demonstrated that participants reported a higher fibre intake at the end of the intervention (4 years) (*n* = 1/1) [82] and a healthier diet pattern in the consumption of unsaturated fat, saturated fat and healthy fat at the end of the intervention (3 years) (*n* = 1/1) [88]. In summary, all studies that investigated a dietary outcome showed an improved dietary outcome.

Concerning physical activity, women in the lifestyle intervention group had higher physical activity, leisure or commuting time activity and exercise at the end of each intervention (*n* = 4/4) [78, 81, 82, 88], a higher exercise capacity at the end of the intervention (6 months), compared to baseline (*n* = 1/1) [85] and higher aerobic activity, flexibility and strength at 6 months follow-up after a three-month intervention, compared to baseline (*n* = 1/1) [77]. In contrast, three studies (*n* = 3/3) revealed no significant differences between physical activity levels between inclusion and after the interventions at 3 months [84], 7 months [76] or during the one-year follow-up after a three-month intervention [87]. Thus, six studies showed a positive impact on physical activity, while three demonstrated no change.

With regards to psychosocial outcomes, the lifestyle interventions led to lower stress perception and higher quality of life at the end of the study (1 year) after a three-month intervention (*n* = 1/1) [87] and less fatalistic and cultural diabetes beliefs at 6 months follow-up after a three-month intervention compared to baseline (*n* = 1/1) [77]. Thus, two studies looked at psychosocial well-being as outcomes and found an improved outcome.

In summary, observational studies demonstrated that there were interactions between lifestyle domains. The studies hint at social support being an important factor for adhering to a healthy lifestyle. Moreover, there were positive relationships between diet and self-efficacy and social support. These two factors were also positively associated with physical activity, more specifically time and intensity were higher when women had higher self-efficacy and social support. The intervention studies demonstrated that most lifestyle interventions improved diet and physical activity, although the effect on physical activity was not sustained in the long term. Lifestyle interventions also augmented psychosocial well-being, but this was only investigated in two studies.

### Clinical outcomes

#### Anthropometric outcomes

Anthropometric outcomes measured in the integrated studies contained BMI, weight, gestational weight gain, waist and hip circumference, body composition and percentage body fat. These outcomes were measured during the postpartum period, except for gestational weight gain, which was measured during pregnancy. In the *observational studies*, only one (*n* = 1/2) study looked at anthropometric outcomes, and more specifically BMI [74]. This study revealed no significant associations between self-efficacy against overeating, and social support from family for diet and BMI, with the exception of a weak correlation between friends’ social support for diet and BMI. After adjustment for the healthy diet index score, dietary self-efficacy and social support were not associated with BMI. The same authors also looked for associations between physical activity-oriented self-efficacy and social support for BMI and found no significant associations between these types of self-efficacy and social support for physical activity and BMI.

Regarding *intervention studies*, 12 interventions (*n* = 12/14) assessed anthropometric outcomes. BMI was reported in eight different studies (*n* = 8/12). This outcome decreased significantly in four studies (*n* = 4/8) at the end of interventions: lasting 3 months [84], 1 year [81], 3 years [88], or 4 years [82]. However, no significant difference was observed in three other studies (*n* = 3/8) at the follow-up measures at 1 year after a three-month intervention [87] and at 6 months after a three-month intervention, compared to baseline [77] and at the end of a six-month intervention, compared to baseline [85]. One study (*n* = 1/8) observed that women following a diet and exercise intervention during pregnancy had a higher pre-pregnancy BMI compared to other groups [83]. The same study (*n* = 1/8) also showed that in women following a lifestyle intervention, BMI increased significantly less between pre- and late pregnancy and between mid and late pregnancy. Waist and/or hip circumference was measured in seven studies (*n* = 7/12) and significantly decreased in five studies (*n* = 5/7), always at the end of the intervention at 3 months [84] or 6 months compared to baseline [85], 1 year [81], 3 years [88] and 4 years [82]. In contrast, two other studies reported no significant change in waist and hip circumference (*n* = 2/7), at the end of a one-year intervention compared to baseline in one study [86] and at 1 year follow-up after a three-month intervention in another study [87]. Participants’ weight was assessed in eight studies (*n* = 8/12). Four studies (*n* = 4/8) revealed a significant decrease in weight after the interventions that lasted 3 months [84], 1 year [81], 3 years [78] or 4 years [82]. However, four other studies (*n* = 4/8) showed no apparent change in weight after interventions that lasted 6 months compared to baseline [85] after a follow-up period of 6 months after a three-month intervention, compared to baseline [77], after a follow-up period of 1 year after a three-month intervention [87] and after a one-year intervention compared to baseline [86]. One study revealed a trend towards reaching the recommended 12-months postpartum weight goal at the end of a 12-month intervention (*n* = 1/1) [76]. One study demonstrated that gestational weight gain was lower at the end of a 7.7 weeks intervention (*n* = 1/1) [80]. In two intervention studies measuring body fat (*n* = 2/2), there was a significant decrease in body fat at the end of the one-year intervention (*n* = 1/2) [81] and at the end of a four-year intervention (n = 1/2) [82]. Another study showed no difference in percent body fat at the end of the six-month intervention, compared to baseline (*n* = 1/1) [85]. In addition, one study showed no change in body composition at the end of the three-month intervention (*n* = 1/1) [84].

In summary, observational studies indicated that social support and self-efficacy had no significant association with BMI. Intervention studies demonstrated a decreased waist and hip circumference and body fat, although the results of lifestyle interventions concerning weight and BMI were inconsistent.

#### Metabolic outcomes

Metabolic outcomes included insulin, glucose, lipid profile, cholesterol, triglycerides, HbA1c, and Apo lipoprotein. None of the *observational studies* assessed metabolic outcomes. Seven of the *intervention studies* (*n* = 7/14) measured metabolic outcomes. Fasting plasma glucose (*n* = 3/7) remained unchanged at the one-year follow-up of a three-month intervention (*n* = 1/3) [87], although it was reduced significantly at the end of two other interventions (*n* = 2/3) that lasted six [79] and 8 weeks [89], respectively. Concerning other glucose-related values (*n* = 3/7), all of these values were reduced in the intervention groups (*n* = 3/3), demonstrating lower one-hour glucose after OGTT at study end (6 weeks) (*n* = 1/1) [79], lower two-hour glucose after OGTT at the 1 year follow-up of a three-month intervention [87] and lower two-hour postprandial blood glucose at the end of an eight-week intervention (*n* = 1/1) [89]. Interestingly, insulin resistance, which was measured in three studies (*n* = 3/7), decreased at the end of a three-year intervention (*n* = 1/3) [88], but no significant change was observed in two other studies (*n* = 2/3) at the end of a three-month intervention [84] or at a one-year follow-up after a three-month intervention [87] (*n* = 2/3). In three studies, HbA1c (glycated haemoglobin) (*n* = 3/7) was measured. It significantly increased between baseline and the six-month follow-up after a three-month intervention compared to baseline in one study (*n* = 1/3) [77] but significantly decreased in the two remaining studies (*n* = 2/3) after a six-week intervention [79] and an eight-week intervention [89]. Three studies measured LDL (low density lipoprotein) –cholesterol (*n* = 3/7); this decreased after a one-year intervention compared to baseline in one study (*n* = 1/3) [86], after a three-year intervention in another [88], and at a six-month follow-up after a three-month intervention compared to baseline in the last study (*n* = 1/3) [77]. Two studies measured HDL (high density lipoprotein)-cholesterol. One study demonstrated a rise in HDL at the end of a one-year intervention compared to the intervention baseline (*n* = 1/2) [86], whilst in the other study it remained the same as during baseline assessments at the six-month follow-up after a three-month intervention (*n* = 1/2) [77]. Two studies measured triglycerides (*n* = 2/7) that decreased in both studies (*n* = 2/2): at a six-month follow-up after a three-month intervention compared to baseline (*n* = 1/2) [77] and at the end of a three-year intervention (*n* = 1/2) [88]. In two separate studies, reductions in total cholesterol were found at a six-month follow-up after a three-month intervention, compared to baseline (*n* = 1/1) [77], and consistency was seen in the lipid profile at the one-year follow-up after a three-month intervention in one study (*n* = 1/1) [87]. Intervention groups had lower fasting plasma insulin levels and Apo lipoprotein at the end of a three-year intervention (*n* = 1/1) [88] and lower plasma insulin levels at the end of a one-year intervention (*n* = 1/1) [81].

In summary, the majority of the studies that included metabolic outcomes revealed a decrease in LDL cholesterol, triglycerides, and in glucose values. Results in HbA1c, insulin resistence and HDL cholesterol were inconsistent and the other outcomes were not measured in enough studies to draw conclusions.

#### Postpartum diabetes status

This outcome was not reported in the *observational studies*. Only two lifestyle *intervention studies* (*n* = 2/14) measured postpartum diabetes status at the end of the intervention (after a three-year intervention in both studies). One intervention study revealed a significant reduction in the risk of diabetes progression (*n* = 1/2) [78]. Another study (*n* = 1/2) [88] showed a 25% decrease in the development of glucose disorders (impaired fasting glucose and impaired glucose tolerance) as well as a 35% decrease in the rate of type 2 diabetes.

In summary, lifestyle interventions led to a reduced risk of postpartum diabetes in the two studies that evaluated this outcome.

#### Delivery and other clinical outcomes

None of the *observational studies* measured delivery or other clinical outcomes. Two of the lifestyle *intervention studies* (*n* = 2/14) measured outcomes related to the delivery, such as macrosomia, adverse pregnancy outcomes, preterm delivery, low birth weight, and caesarean deliveries; two other studies measured other clinical outcomes, such as blood pressure (*n* = 2/14).

In the studies measuring macrosomia (*n* = 2/2), both (*n* = 2/2) demonstrated similar rates of macrosomia in both groups at the end of a 13.2-week intervention [83] and after a 7.7-week intervention [80]. This last study also showed no differences in the rate of adverse pregnancy outcomes [80].

Preterm delivery, low birth weight, and cesarean deliveries were only measured in one study (*n* = 1/1); a significantly decreased risk of preterm delivery and low birth weight at the end of a 13.2-week intervention was found, but there were similar rates of caesarean deliveries compared to a GDM control group [83].

In the studies measuring other clinical outcomes (*n* = 2/14), one study showed a reduction in diastolic blood pressure and no change in systolic blood pressure at the six-month follow-up after a three-month intervention, compared to baseline (*n* = 1/2) [77]. The second study showed that systolic and diastolic blood pressure were unchanged at the end of a one-year intervention compared to baseline [86].

In summary, compared to GDM women in control groups, women in lifestyle interventions showed no differences between the rates of macrosomia, adverse pregnancy outcomes and caesarean section, although there was a decreased risk of preterm deliveries and low birth weight. Concerning results for systolic blood pressure, they were similar throughout groups and time and the results for diastolic blood pressure were inconsistent.

### Quality of studies reviewed

Authors (LG & DYQ) rated the majority of included articles to be of good quality [74–84, 86–89] based on the Joanna Briggs Institute Appraisal Tools (2017) (see Additional file 1: Tables S1 and S2). The checklist for analytical cross-sectional studies [72] was used for the cross sectional observational study [74], the checklist for cohort studies [73] was used for the prospective cohort study [75] whereas for intervention studies, the checklist for randomized controlled trials [70] was employed for the randomized controlled trials [76, 78, 79, 81, 82, 84, 87–89]. For the remaining intervention studies [77, 80, 83, 85, 86], we used the checklist for quasi-experimental studies [71]. Studies rated as having a good quality described in detail the design and methodology used, the process of recruiting participants and the study setting, gave clear and detailed presentation of findings and had study limitations that were unlikely to affect the reliability and validity of study findings. The only study rated as having poor quality [85] did not explain the reasons for drop out in participants and did not conduct analysis to compare the drop outs to the participants remaining in the study. It thus had limited information on data analysis and a small sample size, both of which could lead to a high risk of bias and a poor generalizability of the study.

## Discussion

This integrative review synthesized evidence on the interaction between three different domains: diet (including breastfeeding), physical activity, and psychosocial well-being (including depression, anxiety, sleep, and social support) in women with GDM and their offspring. Moreover, it summarized the effectiveness of interventions addressing more than one lifestyle domain, including diet and physical activity on anthropometric, metabolic, delivery and other clinical outcomes. To the best of our knowledge, this integrative review is the first to synthesize evidence on the relationships and interaction between different lifestyle behaviors, psychosocial well-being, and the efficacy of combined lifestyle interventions in women with GDM and their offspring.

Results from this review indicated that the *interaction between lifestyle domains* produced desirable outcomes. The two observational studies integrated in this review demonstrated that psychosocial well-being such as social support and self-efficacy were important factors associated with adherence to a healthy lifestyle. Indeed, the observational studies demonstrated that social support and self-efficacy were associated to positive changes in diet and physical activity. This is in line with another intervention study showing that psychosocial well-being, such as self-efficacy and social support was positively associated with lifestyle modifications or changes [91]. Similarly, results from the intervention studies showed that lifestyle interventions improved diet and physical activity and augmented psychosocial well-being in study participants, although this last outcome (psychosocial well-being) was only evaluated in two studies. These results underline the importance of apprehending health behavior changes in individuals via more than one domain, thus focusing on a more holistic approach of the individual.

Regarding *anthropometric outcomes,* observational studies demonstrated that psychosocial well-being had no significant association with BMI. This result is not in line with previous research showing that social support and self-efficacy for diet are associated with greater success in weight control [92] and that self-efficacy over dietary behaviours such as emotional eating and dietary restrictions generally lead to healthier weight [93]. This might be due to the fact that only one study investigated this relationship. Results from the intervention studies suggested that most lifestyle interventions achieved successes with regards to waist/hip circumference and body fat. This is in line with previous research demonstrating that diet has an important role to play in weight loss, healthier BMI and other measures of adiposity [94, 95]. Indeed, it is well known that diets setting limits on the intake of energy, trans and saturated fat, and/or energy from carbohydrate and increased fiber intake help GDM women with weight management [2]. Physical activity might also play a role in the relationship between lifestyle interventions and an improvement in anthropometric outcomes, as studies also suggest that physical activity is associated with positive changes in eating self-regulation and may lead to healthy eating. In particular, it improves psychosocial well-being and could prevent emotional eating, consumption of foods high in calories, and binge eating [96]. Higher adherence to physical activity could therefore increase eating self-regulation and may lead to lower anthropometric outcomes such as weight, BMI and waist circumference measures. Even though the results of lifestyle intervention studies led to decreases in some anthropometric outcomes, weight and BMI demonstrated inconsistent results. This might partly be due to the diversity of diet and physical activity interventions, as well as the length of the studies and adherence to the intervention.

Regarding *metabolic outcomes*, the intervention studies led to a decrease in LDL cholesterol, triglycerides and glucose values compared to the control groups, although results for HbA1c, insulin resistence and HDL cholesterol were inconsistent. For the decreasing outcomes, the diet component of the intervention studies might have had an impact on these findings. Indeed, previous research has shown that the high dietary fiber intake may reduce appetite and food consumption, delay gastric emptying, slow food digestion and absorption [97]. This should have led to a decrease glucose absorption and also plasma insulin levels [98]. Our results are in line with these findings, as three interventions measured glucose values and two studies lead to improvements in two measured glucose values. The third study led to improvements in two-hour glucose after OGTT and to similar results in fasting plasma glucose. Research shows that the consumption of a DASH diet leads to a decrease in lipids and fasting glucose, as it has a positive impact on the lipid profile in women with GDM [99], as well as in other populations [100, 101]. In our review, the Mediterranean diet was associated with overall improved metabolic health outcomes. In pregnancy, these diets may have protective benefits for overweight and obese women who are at risk for both short and long-term metabolic outcomes [102]. The physical activity component of the intervention studies might have also played a role in the improvements of some of these metabolic outcomes. Indeed, previous research has shown that regular exercise increases insulin action by stimulating glucose uptake in the muscle through glucose transport proteins (GLUT4) that mediate insulin-dependent glucose uptake [103], and our results showed improvements in 2.5/3 of the studies analyzing glucose as an outcome. A meta-analysis of randomized controlled trials in women with GDM showed that exercise significantly improved postprandial glucose and lowered fasting blood glucose [104]. It was therefore not surprising that participants who had lifestyle interventions had lower fasting plasma insulin levels and two-hour postprandial blood glucose than those in the control group. Results for HDL cholesterol, HbA1c and insulin resistence were inconsistent in the intervention studies. This might be explained by the fact that the interventions were probably not intense enough to have a long term impact on these outcomes. Another explanation could be the low adherence to the intervention regime. Overmore as HDL is also influenced by oestrogen status, it might be a strong confounder for this outcome in this population and might have impacted these results [105].

Two intervention studies showed reductions in the rate of *postpartum diabetes status,* [78, 88]. In a systematic review that examined the cumulative incidence of type 2 diabetes in women with GDM, the progression to type 2 diabetes after GDM increased steadily within the first 5 years after delivery [106]. According to Tobias et al., diet plays a role in the reduction of postpartum diabetes status, as higher adherence to a Mediterranean diet was associated with a 40% lower risk of diabetes compared to those in the lower adherence group in their cohort study [107]. In the same study, similar risk reductions were observed for the DASH diet, even after multiple adjustments of covariates [107]. Elevated fasting glucose and HbA1c levels during pregnancy may be associated with a more pronounced progression to diabetes after GDM [108–110]. Adherence to a lifestyle intervention designed to lower weight gain and improve metabolic health during pregnancy may prevent the development of postpartum diabetes, as observed in this review. Physical activity has also been implicated in the prevention or delay in postpartum diabetes in women with GDM [111]. A prospective cohort study recently showed that women with GDM within the Nurses Health Study II cohort had a 9% reduced risk for postpartum diabetes for every 100 min of moderate intensity physical activity. Interestingly, an increase of 150 min per week of moderate intensity physical activity led to a 47% lower risk of diabetes after GDM [36].

Regarding *delivery and other clinical features,* the results of one study demonstrated a decrease in preterm delivery rates and low birth weight. Regarding preterm delivery, this outcome can be caused by various pre-existing conditions in the mother [112] and thus might not depend on lifestyle interventions. Concerning low birth weight, one of the studies found fewer low birth weight after a 13.2-week intervention [83]. Thus, our results are not in line with a previous systematic review and meta-analysis of randomized controlled trials of dietary interventions in women with GDM showing that dietary interventions were associated with lower birth weight compared with controls [113]. One explanation could relate to the fact that women in the integrated study might not have all received the same type of lifestyle intervention. Indeed this study mentioned that the lifestyle interventions were retrospectively auto-reported by questionnaire [83]. Finally, we found similarities in the rates of macrosomia, in the intervention studies in the control as well as intervention groups [80, 83]. Thus, our results are in line with the findings of a recent review indicating that diet and/or physical activity interventions lead to a similar risk of macrosomia compared to a control group in overweight and obese women [114]. Previous research has shown that macrosomia, adverse pregnancy outcomes and caesarean sections are dependent on a number of different factors and/or on the maternal diabetes status [1, 115, 116] and thus, lifestyle interventions might have little to no effect on these outcomes. The results for systolic blood pressure were similar between baseline and at 6 months follow-up after a three-month intervention [77] and similar compared to baseline in an other study [86]. Finally, for diastolic blood pressure, our results were inconsistent. These results are comparable with previous research showing no difference in systolic and diastolic blood pressure between different control groups and GDM women [117], except for one of the integrated studies demonstrating a decrease in diastolic blood pressure at 6 months follow-up after a three-month intervention, compared to baseline [77].

**Fig. 2:**
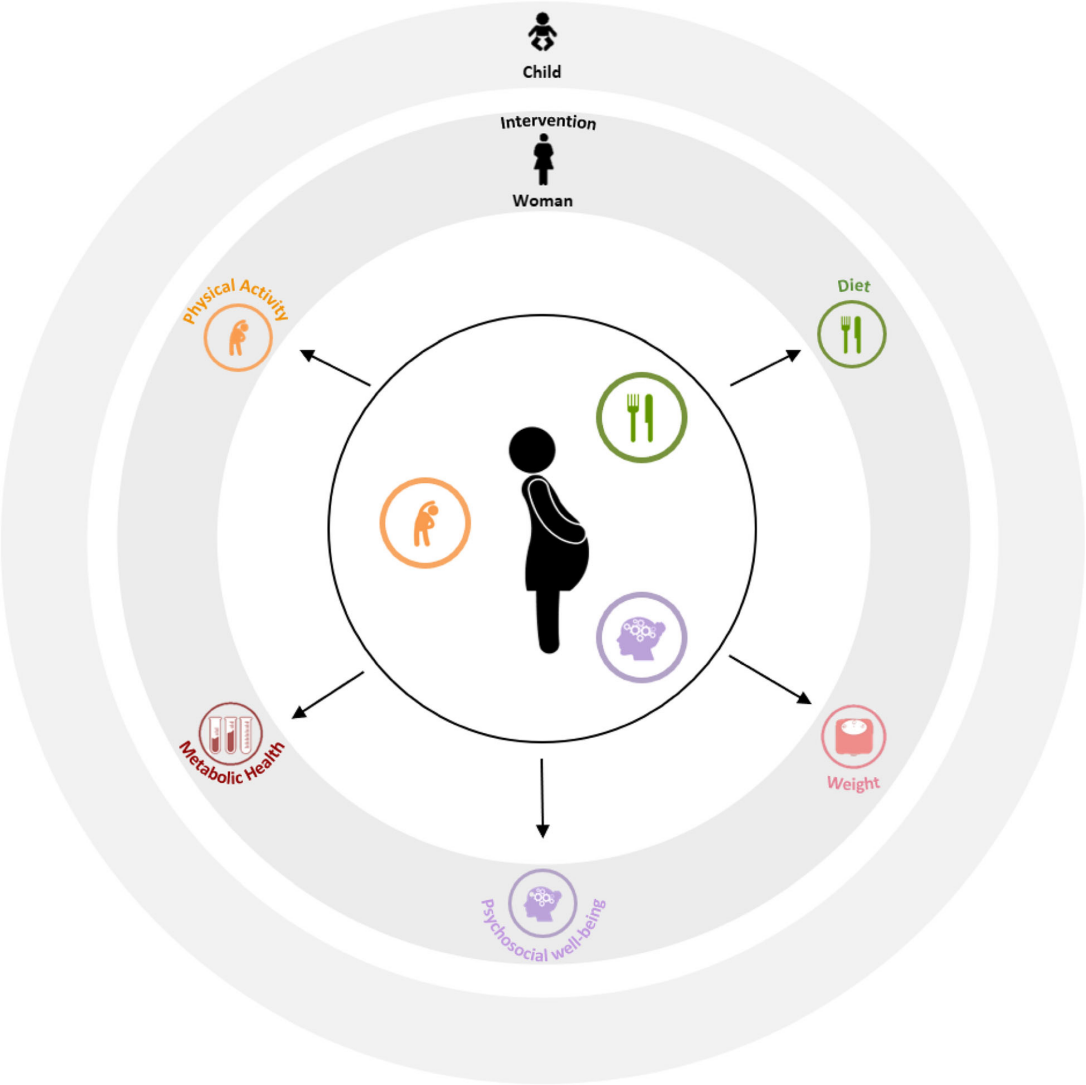
Integrative review model. Conceptual model resulting from the integrative review proposes that interventions targeted at mitigating the risks associated with a GDM pregnancy should not only include diet and physical activity domains but may also integrate and/or include strategies for improving self-efficacy and self-regulation of eating, exercise, psychosocial well-being, and social/ family support. In the first black circle, diet, physical activity, and psychosocial well-being interventions for women with GDM are represented. In the second gray circle, the outcomes which are improved for the mother following a diet, physical activity and psychosocial intervention are illustrated. Finally, the largest gray circle represents the neonatal outcomes which may also be improved if the mother follows a diet, physical activity and psychosocial intervention

Overall, evidence from this integrative review suggests that *lifestyle interventions* including a psychosocial intervention during pregnancy could augment the women’s adherence to diet and physical activity, which in turn might have complementary and interactive effects on the physiological and psychological health of women with GDM. We therefore propose that combined diet, physical activity, and psychosocial interventions could positively influence physiological and psychological processes toward healthy outcomes (Fig. 2) and should be tested. Arguments that cognitive-behaviorally supported exercises, self-efficacy and social support can facilitate changes in eating behavior through associated psychological changes have emerged, also outside of pregnancy. This is partly because diet and physical activity domains of a lifestyle intervention may also benefit from improved psychosocial outcomes. Thus, exercise during pregnancy can influence physiological processes, such as energy metabolism and appetite, as well as psychological factors, including self-efficacy, body image, or mood [118, 119]. The interactive mechanisms of these factors could lead to stronger motivation and confidence, which could improve adherence to physical activity. Long-term exercise adherence, as well as eating self-regulation and dietary compliance may also result in gestational weight gain control, improved metabolic outcomes, and again higher levels of psychosocial well-being during pregnancy and in the post-partum period. On the other hand, psychosocial vulnerability (including depression, stress, and lack of social support), lack of diet self-regulation and physical inactivity may negatively influence birth outcomes, including caesarean deliveries, macrosomia and other infant physiological disorders, such as hypoglycemia, as well as adverse outcomes in the mother during the post-partum period [90, 120–122]. According to our results and proposed model (see Fig. 2), interventions targeted at mitigating the risks associated with a GDM pregnancy should not only include diet and physical activity domains but may also integrate and/or include strategies for improving self-efficacy and self-regulation of eating, exercise, psychosocial well-being, and social/ family support. After all, the success of a combined diet and exercise intervention may also depend on the mothers’ psychosocial well-being (depression, stress, self-efficacy and social support) during pregnancy.

### Strengths and limitations

This integrative review has many strengths. This study followed the PRISMA guidelines as well as Whitemore and Knafl’s recommendations We used a comprehensive search strategy and independent reviewers carried out identification of relevant studies. The majority of our included studies were of RCT design with large sample sizes and follow-up periods. We also included psychosocial well-being and focused on combined interventions, which, to our knowledge, has not been done before. Nevertheless, some limitations need to be addressed. Firstly, conducting an integrative literature review lead to integrating studies with large heterogeneities regarding the intervention and follow-up periods across studies, as well as in the types of lifestyle interventions used in each individual studies. Thus, our results need to be interpreted with caution. In addition, psychosocial well-being was only investigated in observational studies, even though it was assessed in intervention studies as an outcome. Moreover, although we had also searched for terms, such as depression, anxiety and sleep in the psychosocial well-being domain, no results were found for these outcomes. This might be due to the fact that, as mentioned, psychosocial well-being was only present in two observational studies and as an outcome in two intervention studies. Furthermore, although we had also screened for articles for parenting, we found no results concerning the partner except in observational studies. Indeed, in the observational studies, the partners appeared as “social support from family” but no other results were found. The different components of the lifestyle interventions and types of diet and physical activity as well as the approach and the patient population may account for the differences in study results and conclusions. In addition, the inability of the lifestyle interventions to account for or adjust for individual attitudes and behaviors, particularly psychosocial factors, might have influenced the results of these studies. This is because positive results on changing diet and physical activity habits are often related to self-efficacy or social support, as seen in the observational studies. Finally, the issue of publication bias can be a limitation to this study, as studies reporting no significant results are rarely published [123].

### Clinical implications and future directions

The findings of this integrative literature review reveal that diet, physical activity, and psychosocial well-being relate and interact in women with GDM. On the one hand, diet and physical activity were associated with psychosocial well-being. On the other hand, this review showed that psychosocial well-being, such as self-efficacy and social support may be important when adopting a healthy diet and physical activity habits. Thus, we propose that any intervention focusing on behavioral change, should evaluate and consider integrating psychosocial well-being as part of the intervention components, as this might add to the lack of research in this domain. Even though diet and physical activity interventions may reduce some of the risks associated with GDM, the findings of this integrative review suggest that there may be merit in further exploring the option of psychosocial well-being in future interventions. This may increase patients’ willingness to change attitudes and inform positive behavioral changes that would expand the current scope of strategies in reducing the risk associated with GDM. Future studies that plan to adopt psychosocial interventions should focus on self-efficacy and/or social support, as both elements are associated with diet and physical activity habits. However, this might not be easy, as it implies that women already have a support system on which they can rely to help them change their behavior and that self-efficacy can be improved in this life period within a lifestyle intervention. It is also known that prenatal maternal stress exposure and stress perception are associated with less favorable obstetric outcomes, such as caesarean section [90, 120, 121]. Thus, future interventions may focus on the psychosocial well-being of women with GDM to help alleviate and/or ameliorate stress symptoms [124]. Furthermore, partners of women could also be integrated as social support for women with GDM that need to make lifestyle changes. Finally, it would also be interesting to conduct a review on qualitative studies to identify participant perception and lived experiences with lifestyle interventions in women with GDM in order to fine-tune future interventions.

## Conclusion

This integrative review showed that diet, physical activity and psychosocial well-being interact in women with GDM. We found that lifestyle interventions led to a better dietary quality in all studies, improvements in physical activity in more than half of the studies measuring this outcome, lower stress perception, higher quality of life, less fatalistic and cultural diabetes beliefs, some better anthropometric and metabolic health outcomes, lower rates of diabetes progression following GDM and to less preterm deliveries and a higher birth weight. The observational studies also demonstrated the importance of social support and self-efficacy in relation to a healthy lifestyle in women with GDM. Given that psychosocial well-being, such as social support and self-efficacy, are associated with physical activity and healthy dietary choices, we recommend that future intervention studies consider integrating psychosocial well-being in a combined diet and physical activity intervention to investigate the role of self-efficacy and social support on GDM.

## Additional files

Additional file 1: This additional file contains** Table S1** and **Table S2** which give a summary of the observational and intervention articles integrated in the literature review. Both tables give information about the authors, year and country of publication, the design, sample and objective of each study, the selection criteria applied to the study participants, information on how diet, physical activity and psychosocial well-being were assessed, and finally the quality of the study, appraised through JBI criteria. In the tables describing the observational studies; the major findings of each study are summarized. In the tables describing the intervention studies; the type of intervention as well as the results concerning the intervention group are summarized. A legend describing abbreviations or symbols can also be found below each table. (DOCX 40 kb)

Additional file 2: Full Search Strategy. This additional file contains the precise comprehensive search strategy used in this integrative literature review, thus it includes the terms we searched for in each database: CINAHL, PsycINFO, Embase, Pubmed, and Cochrane. This file also specifies the number of articles found in each data base for both research periods. (DOCX 17 kb)

## Abbreviations

GDM: Gestational diabetes mellitus
OGTT: Oral glucose tolerance test
