# Supplementary material for: How diet, physical activity and psychosocial well-being interact in women with gestational diabetes mellitus: an integrative review
Source: BMC Pregnancy Childbirth. 2019 Feb 7;19:60. doi: 10.1186/s12884-019-2185-y (PMC6367798; doi:10.1186/s12884-019-2185-y)
Supplement: Supplementary file 1 — This additional file contains Table S1 and Table S2 which give a summary of the observational and intervention articles integrated in the literature review. Both tables give information about the authors, year and country of publication, the design, sample and objective of each study, the selection criteria applied to the study participants, information on how diet, physical activity and psychosocial well-being were assessed, and finally the quality of the study, appraised through JBI criteria. In the tables describing the observational studies; the major findings of each study are summarized. In the tables describing the intervention studies; the type of intervention as well as the results concerning the intervention group are summarized. A legend describing abbreviations or symbols can also be found below each table. (DOCX 40 kb) [file 12884_2019_2185_MOESM1_ESM.docx]

**Table S1. *Summary of observational articles integrated in the review.***

| **Authors (year)**  **Country** | **Study design**  **Sample**  **Objective** | **Selection criteria** | **Diet, physical activity & psychosocial assessment** | **Major findings** | **Quality**  JBI quality appraisal tools |
| --- | --- | --- | --- | --- | --- |
| Kim et al. (2008)  U.S.A | Cross-sectional  n=228  To examine the associations between two potential facilitators of healthy behaviors (self-efficacy and social support) on diet, physical activity and BMI among women with histories of GDM | **Inclusion**  Women with GDM within the past 5 years and with ≥ 1 health-care utilization event during the past year  **Exclusion**  Women with T1DM or T2DM before their pregnancy, denied having had GDM, were currently pregnant without GDM or unable to give informed consent | **Diet**  Weight Efficacy Lifestyle Questionnaire  **Physical Activity**  5-item scale  **Psychosocial**  Scale | Self-efficacy and social support from family and friends was associated with better dietary scores and physical activity.  No significant associations existed between psychosocial constructs and BMI. | Good |
| Kaiser et al. (2016)  Switzerland | Prospective Cohort Study  n=173  To specify the determinants of postpartum physical activity and dietary habits after a pregnancy complicated by GDM in a population of Swiss women | **Inclusion**  Women with diagnosis of GDM in the current or in a previous pregnancy without diagnosis of T1DM or T2DM;  ≥ 18 years; if they could read, write, and speak French. | **Diet & Physical activity**  Rapid Eating and Activity Assessment for Participants, short version questionnaire  **Psychosocial**  Social support and self-efficacy scale | Multivariate regression analysis found that Lower level of social support (p<0 .001) and more perceived barriers to a healthy lifestyle (p = 0.002) were determinants in a low adherence to healthy lifestyle in the postpartum period after GDM | Good |

**Legend**: BMI: Body mass index; GDM: gestational diabetes mellitus; T1DM: type 1 diabetes mellitus; T2DM: type 2 diabetes mellitus.

**Table S2. *Summary of Intervention studies integrated in the review.***

| **Authors (year)**  **Country** | **Study design**  **Sample**  **Objective** | **Selection criteria** | **Diet & physical activity**  **assessment** | **Intervention** | **Major findings in intervention group** | **Quality**  JBI quality appraisal tools |
| --- | --- | --- | --- | --- | --- | --- |
| O’Dea et al. (2015)  Ireland | RCT  n=50  To evaluate a 12-week group-based lifestyle intervention program for women with pre-diabetes following GDM | **Inclusion**  IFG, or IGT, or IR and at least two CV risk factors  **Exclusion**  T2DM, current pregnancy, insufficient English language fluency | **Diet**  Mediterranean diet score  **Physical Activity**  Self-reported | **Starting in the postpartum period**  **Diet and Physical Activity**  12-week intensive lifestyle program, delivered by a multidisciplinary team, consisting of 2.5 h/wk spread over a group exercise program, a group education seminar, and a one-to-one session involving a motivational interview and individual goal setting | **At the end of the study, at 1-year follow-up, after a three months intervention, compared to control group**  ↗ (trend) diet adherence, diet self-efficacy  = PA levels  ↙ stress perception, ↗ quality of life  = BMI, weight, waist circumference  ↙ 2hr glucose after OGTT  = Insulin resistance, FPG and lipid profile | Good |
| Ferrara et al. (2011)  U.S.A. | RCT  n=197  To pilot the feasibility of a prenatal/postpartum intervention that aimed to reduce diabetes risk factors among women with GDM | **Inclusion**  Women with GDM  **Exclusion**  Aged< 18; multiple gestation; diabetic retinopathy; high-risk pregnancy, thyroid diseases diagnosed in the last 30 days, non-English speaker. | **Diet**  FFQ  **Physical Activity**  Interview questionnaire | **Starting during pregnancy**  The intervention program aims to modify **diet and Physical Activity** in 3 phases:  **Prenatal phase**  Encourage women to follow the ADA diet and engage in moderate intensity PA for 150 min/wk  **Postpartum phase**  Encourage women to perform 150 min of moderate or harder PA/wk and to consume ≤ 25% of total calories from fat/day  **Maintenance phase**  Reinforce the positive behavioral changes achieved and address relapse | **7 months postpartum, compared to control group**  ↙ fat intake, ↗ (trend) proportion of women who partially or exclusively breastfed  = PA levels  **At the end of the study, at 12 months postpartum, compared to control group**  ↗ (trend) proportion of women who reached the PP weight goal. The intervention was more effective among women who did not exceed the recommended GWG | Good |
| Peacock et al. (2015)  Australia | RCT  n=31  To develop a program to support behavior changes in diet and physical activity for women with a history of GDM and BMI to delay or prevent T2D | **Inclusion**  Women with GDM who had been diagnosed and treated for GDM, with a self-reported BMI >25 kg^.^m^-2^  **Exclusion**  Women currently pregnant, T2DM, not fluent in English, using hypoglycaemic medications | **Diet**  Fibre Index, Health and Wellbeing Self Efficacy Survey  **Physical Activity**  Interview questionnaire (Australian Women’s Activity Survey) | **Starting in the postpartum period**  **Diet (nutrition coaching)**  Four one-hour group sessions to facilitate behavior change  **Physical Activity**  Weekly goals (web-based) were generated based on the previous weeks steps recorded with a pedometer. The goals were gradually increased, until the maximum of 10’000 steps/d was reached | **At the end of the study, at 3 months, compared to control group**  ↗ self-efficacy related to food choices  = PA  ↙ weight, BMI, hip and waist circumference  = body composition  = HOMA-IR | Good |
| Philis-Tsimikas et al. (2014)  U.S.A. | Interventional  n=84  To evaluate the effectiveness of a lifestyle intervention to reduce T2DM and CVD risk among low income Latinas with a history of GDM | **Inclusion**  Latinas with GDM in the past 3 years  **Exclusion**  Women with T2DM, who were pregnant, and/or who had a serious health condition | **Diet**  Questionnaire (Food Screener)  **Physical Activity**  Questionnaire (rapid assessment of physical activity) | **Starting in the postpartum period**  The intervention was adapted from the DPP  **Diet and Physical Activity**  Achieve and maintain a weight reduction of at least 7% of initial body weight, and to achieve and maintain a level of PA of at least 150 min/wk through moderate intensity activity | **At the end of the study, at 6 months follow-up after a 3 months intervention, compared to baseline**  ↙ dietary fat intake  ↗ aerobic activity, ↗ flexibility/strength  ↙ fatalistic, ↙ diabetes-specific cultural beliefs  = weight and BMI  ↙ Total cholesterol; ↙ LDL, =HDL, ↙ TG; ↗ HbA1c  = systolic blood pressure, ↙ diastolic blood pressure | Good |
| Ratner et al. (2008)  U.S.A. | RCT  n=2190  To identify individuals with IGT and intervene in an effort to prevent or delay their progression to diabetes  To examine the differences between women with and without a reported GDM | **Inclusion**  Women with a history of GDM aged ≥ 25, BMI ≥ 24 kg^.^m^-2^ (≥ 22 for Asian-Americans), FPG of 95–125 mg/dL, 2-h glucose 140-199 mg/dL in the OGTT  **Exclusion**  Recent myocardial infarction, symptoms of coronary heart disease, serious illness, or use of medications known to impair glucose tolerance. | **Diet**  Semiquantitative FFQ  **Physical Activity**  Standardized questionnaires | **Starting in the postpartum period**  The intervention group followed the DPP  **Diet and Physical Activity**  Achieve and maintain a weight reduction of at least 7% of initial body weight, and to achieve and maintain a level of PA of at least 150 min/wk through moderate intensity activity | **At the end of the study, at 3 years, compared to control group**  ↙ weight  ↗ of PA (~1.5h/wk) at 1 yr follow-up; not sustained in women with a history of GDM at 3 yr follow-up  ↙ risk of progression to T2DM | Good |
| Pérez-Ferre et al. (2015)  Spain | RCT  n=237  To evaluate the efficacy of a lifestyle intervention for the prevention of glucose disorders in women with prior GDM | **Inclusion**  Women diagnosed with GDM.  **Exclusion**  Impaired fasting plasma glucose (≥ 100 mg/dL) in the first postpartum evaluation and plan for new pregnancy during the three years of follow-up. | **Diet and Physical Activity**  Semiquantitative questionnaire (Lifestyle questionnaire) | **Starting in the postpartum period**  **Diet**  Adherence as much as possible to the Mediterranean diet  **Physical activity**  4x/week, two days at the hospital and two days at home with duration of 50-60 min during 10 weeks between 3-6 months post-delivery. The sessions consisted in progressive aerobic activities and muscular conditioning performed at moderate intensity | **At the end of the study, at 3 years, compared to control group**  ↗ nutrition pattern, healthier pattern in the consumption of unsaturated fat, saturated fat and healthy fat  ↗ PA pattern as in the control group  ↙ BMI; ↙ waist circumference  ↙ fasting plasma insulin; ↙ HOMA-IR; ↙ LDL-cholesterol; ↙ TG; ↙ Apo lipoprotein B  ↙ of ~25% in the conversion rate to glucose disorder and ↙ of 35% in the conversion rate to T2DM | Good |
| Rautio et al. (2014)  Finland | Interventional  n=265  To compare cardio-metabolic risk profile and responses to a 1-year lifestyle intervention program in women with and without history of GDM | **Inclusion**  Women with GDM, IFG, IGT or coronary heart disease  **Exclusion**  NA | **Diet and Physical Activity**  Questionnaire | **Starting in the postpartum period**  **Physical Activity & Diet**  Mainly based on the principles of empowerment developed during exercise or weight maintenance groups, lectures or individual counseling | **At the end of the study, at 1 year, compared to baseline**  = weight, waist circumference  ↗ HDL-cholesterol, ↙ LDL-cholesterol  = systolic & diastolic blood pressure | Good |
| Jovanovic-Peterson et al. (1989)  U.S.A. | RCT  n=19  Evaluate the  impact of a training program on glucose tolerance in  GDM | **Inclusion**  Women with GDM  **Exclusion**  NA | NA | **Starting during pregnancy**  **Diet**  Control & intervention groups followed a 6 weeks of standards diet containing 40% of CHO, 20% protein & 40% fat. Energy requirement was calculated at 24-30 kcal/kg/24h divided in 3 meals and 3 snacks  **Physical Activity Intervention**  Exercise program of 6 weeks' duration that consisted of 20 min of supervised aerobic training. The patients were monitored for 20 min of cardiovascular work during which they maintained their target heart rate, which was calculated to be equal to (220 - age) x 70%. As each patient adapted to her workload in terms of perceived exertion and heart rate, the workload (or resistance) (kg x meters x min-I) was increased or decreased by 10% to 20%; thus maternal heart rate was maintained in the training range. The exercise session never exceeded 50% maximal oxygen consumption V0_2_max in any case. 50% oxygen consumption was estimated based on their previous work that related maternal heart rate in beats/min to the V02 at steady state utilizing a metabolic cart (Beckman model S/N 614, D/N 11265, Fullerton, Calif.) | **At the end of the study, at 6 weeks, compared to control group**  ↙ HbA1c, FPG, 1hr glucose after OGTT | Good |
| Hu et al.  (2012)  China | RCT  n=1180  To assess whether lifestyle intervention can reduce type 2 diabetes risk in women with prior GDM in the Tianjin GDM Prevention Program | **Inclusion**  Aged 20–49, women with GDM between 2005 and 2009.  **Exclusion**  Aged <20 or ≥50, at the screening visit: FPG ≥7.0 mmol/l or 2-h glucose ≥ 11.1 mmol/l in the OGTT, taking medicines known to alter OGTT, presence of any chronic diseases, currently pregnant, planning to become pregnant in the next 2 yrs | **Diet**  5 x 3-day 24-h food records; questionnaire  **Physical Activity**  Questionnaire | **Starting in the postpartum period**  **Diet**  Reduction of at least 10% of total calories of their normal meals to lose 5–10% of initial body weight in women with BMI ≥ 24 kg/m^2^  Consume <30% of energy from total fat, <10% of energy from saturated fat, 55-65% of energy from carbohydrate, 20–30 g/d of fiber  **Physical Activity**  Gradually increase the physical activity to reach 30 min/day in moderate or vigorous physical activity 7 d/wk | **At the end of the study, at 1 year, compared to control group**  ↗ leisure time activity  ↙ weight, ↙ BMI, body fat, waist circumference  ↙ plasma insulin level | Good |
| Artal et al.  (2007)  U.S.A. | Interventional  n=96  To assess whether weight-gain restriction regimen, with or without exercise, would impact glycemic control, pregnancy outcome, and total pregnancy weight gain in obese subjects with GDM | **Inclusion**  < 33 GA, BMI > 25 kg/m^2^, not yet managed with insulin, aged > 18  **Exclusion**  ACOG contraindication to exercise | NA | **Starting during pregnancy**  **Diet**  Meal plan was prescribed for both group by a registered dietitian according the energy needs calculated on the prepregnancy BMI and which content 40-45% of carbohydrates.  **Physical Activity**  Moderate exercise program not to exceed the 60% VO_2 max_. Encouraged to exercise once a week by walking on a treadmill or by riding a semi recumbent cycle ergometer based on an exercise prescription under the supervision of an exercise routine on the remaining 6 days/week at home | **At the end of the study, at a calculated mean of 7.7 weeks, compared to control group**  ↙ GWG  = macrosomia, adverse pregnancy outcome | Good |
| Youngwanichsetha et al.  (2014)  Thaïland | RCT  n=170  To investigate the effect of mindfulness eating and yoga exercise on blood sugar levels among pregnant women with GDM | **Inclusion**  Pregnant Thai women diagnosed with GDM with 24–30 wk GA, FPG < 105 mg/dl, postprandial blood glucose < 120 mg/dl, not receiving insulin therapy, having no serious complications  **Exclusion**  NA | NA | **Starting during pregnancy**  8 weeks to perform mindfulness eating and yoga exercise  **Diet**  Mindfulness eating composed of five steps: 1) setting a goal for blood glucose control, 2) integrating medical nutrition therapy including carbohydrate choices and low glycemic index food, 3) considering portion size, 4) being aware while consuming diabetic food, and 5) eating slowly for 30-45 min.  **Physical activity**  Yoga exercise at home five times a week using deep-breathing techniques and posture and movements. It was designed for 15–20 minutes daily practice, corresponding to 9 postures repeated for ten times | **At the end of the study, at 8 weeks, compared to control group**  ↙ FPG, 2hr postprandial blood glucose and HbA1c | Good |
| Mukerji et al. (2015)  Canada | Interventional  n= 17  To assess the feasibility and effectiveness of a 6-month customized, home-based lifestyle program for women with recent GDM | **Inclusion**  Women aged > 18 with GDM and prepregnancy BMI ≥ 25 kg^.^m^-2^  **Exclusion**  Not english speaking, prepregnancy T1DM or T2DM, pregnant again, significant medical or fetal complications | **Physical Activity**  Weekly 15-minute telephone calls | **Starting in the postpartum period**  **Diet**  General diet counselling using Canada’s Food Guide  **Physical Activity**  A personalized home-based exercise program, incorporating the participants’ baseline fitness, and current exercise habits. All women were counselled to meet 150 min of moderate aerobic exercise/wk and to keep an exercise log/wk. Weekly telephone calls were scheduled with an exercise specialist to provide coaching, review adherence to exercise logs, advance goals and address barriers | **At the end of the study, at 6 months, compared to baseline**  ↗ exercise capacity  ↙ waist circumference  = weight, BMI, % body fat | Poor |
| Liu et al.  (2018)  China | RCT  n=1180  To report the weight loss findings after the first year of a lifestyle intervention trial among women with GDM | **Inclusion**  Women aged 20–49, with GDM between 2005 and 2009.  **Exclusion**  Aged <20 or ≥50, at the screening visit: FPG ≥7.0 mmol/l or 2-h glucose ≥ 11.1 mmol/l in the OGTT, taking medicines known to alter OGTT, presence of any chronic diseases, currently pregnant, planning to become pregnant in the next 2 years | **Diet**  5 x 3-day 24-h food records; questionnaire  **Physical Activity**  Questionnaire | **Starting during pregnancy**  **Diet**  Each participant met one-on-one with a dietician who instructed the participant on how to achieve several goals: reduction of 5% to 10% of initial body weight in women with BMI ≥24 kg/m2 by reducing at least 10% of total calories in their normal meals; total fat intake <30% of energy consumed; carbohydrate intake 55% to 65% of energy consumed; fiber intake 20-30 g/d  **Physical Activity**  During the first 4wk of intervention, the level of PA increased to at least 30 min of moderate-to-vigorous PA per day, 7 days/wk; and was then maintained during the entire period  Each participant completed a questionnaire on changes in major dietary and PA habits from the last visit, and 3-day 24-hour food records 5 times during the first year for assessment by the dietician. 2 phone calls were performed during the first year to encourage compliance to intervention | **At the end of the study, at 4 years, compared to control group**  ↗ fibre intake  ↗ commute and leisure time activity, exercise  ↙ weight, BMI, waist circumference, body fat | Good |
| Wang et al.  (2015)  China | Interventional  n=14’168  To evaluate whether exercise intervention can be applied to pregnant women with GDM for controlling gestational weight gain and combating GDM-related outcomes. | **Inclusion**  NA  **Exclusion**  pre-existing diabetes, multiple births, and missing data on major items | **Diet and Physical Activity**  Individual interview | **Starting during pregnancy**  **Diet**  Reduce intake of sugar, eat more vegetables, reduce fat intake, and the total energy intake 1800 kcal/j  **Physical Activity**  Sit less, take more steps, be more active, incorporate light and moderate PA as much as possible into their daily life. | **At the end of the study, at a calculated mean of 13.2 weeks, compared to control group**  ↙ BMI increase between pre and late-pregnancy and between mid and late-pregnancy  ↙ risk of preterm birth, low birth weight  = macrosomia, caesarean | Good |

**Legend:**

↙: significant reduction, ↗: significant augmentation; =: no significant difference; BMI: body mass index; CVD: cardiovascular disease; DBP: diastolic blood pressure; DPP: diabetes prevention program; FFQ: Food Frequency Questionnaire; FPG: fasting plasma glucose; GDM: gestational diabetes mellitus; GWG: gestational weight gain ; HbA1c : glycosylated hemoglobin; HOMA-IR: homeostasis model assessment-insulin resistance; IFG: impaired fasting glucose; IGT: impaired glucose tolerance; ILS: intensive lifestyle intervention; OGTT: oral glucose tolerance test; PA: physical activity; RCT: randomized control trial; SES: socioeconomic status; T2DM: type 2 diabetes mellitus; TG: Triglycerides
