# Supplementary material for: How diet, physical activity and psychosocial well-being interact in women with gestational diabetes mellitus: an integrative review
Source: BMC Pregnancy Childbirth. 2019 Feb 7;19:60. doi: 10.1186/s12884-019-2185-y (PMC6367798; doi:10.1186/s12884-019-2185-y)
Supplement: Supplementary file 2 — Full Search Strategy. This additional file contains the precise comprehensive search strategy used in this integrative literature review, thus it includes the terms we searched for in each database: CINAHL, PsycINFO, Embase, Pubmed, and Cochrane. This file also specifies the number of articles found in each data base for both research periods. (DOCX 17 kb) [file 12884_2019_2185_MOESM2_ESM.docx]

**Full search strategy**

**PubMed**

(("Diabetes, Gestational"[Mesh]) AND (("Exercise"[Mesh] OR "Physical Fitness"[Mesh] OR "Motor Activity"[Mesh]OR "Bicycling"[Mesh]) OR ("Feeding Behavior"[Mesh] OR "Hunger"[Mesh] OR "Satiation"[Mesh] OR "Dietary Fats"[Mesh] OR "Diet"[Mesh] OR "Dietary Carbohydrates"[Mesh] OR "Body Weight"[Mesh] OR "Body Composition"[Mesh] OR "Waist Circumference"[Mesh]) OR ("Life Style"[Mesh] OR "Attitude to Health"[Mesh] OR "Health Behavior"[Mesh] OR "Depressive Disorder"[Mesh] OR "Depression"[Mesh] OR "Stress, Psychological"[Mesh] OR "Parenting"[Mesh] OR "Parent-Child Relations"[Mesh] OR "Anxiety"[Mesh:NoExp] OR "Anxiety Disorders"[Mesh] OR "Postpartum Period"[Mesh])) NOT ("animals"[mh] NOT "humans"[mh])) OR ((Pregnancy-Induced Diabete*[tiab] OR Gestational diabete*[tiab]) AND (Exercise[tiab] OR Swimming[tiab] OR Stretching[tiab] OR Walking[tiab] OR Physical activit*[tiab] OR Aerobic[tiab] OR "Strength training"[tiab] OR Sedentary[tiab] OR inactivity[tiab] OR Cycling[tiab] OR running[tiab] OR resistance training[tiab] OR feeding behav*[tiab] OR eating behav*[tiab] OR Feeding Pattern*[tiab] OR Eating Pattern*[tiab] OR Food habit*[tiab] OR Eating habit*[tiab] OR Diet habit*[tiab] OR Dietary habit*[tiab] OR Breast feeding[tiab] OR Breastfeeding[tiab] OR (Mindful*[tiab] AND eating[tiab]) OR Intuitive eating[tiab] OR Food intake[tiab] OR Hunger[tiab] OR satiation[tiab] OR dietary fat*[tiab] OR diet[tiab] OR diets[tiab] OR sugar*[tiab] OR carbohydrate*[tiab] OR sucrose[tiab] OR weight[tiab] OR overweight[tiab] OR obesity[tiab] OR body composition[tiab] OR appetite[tiab] OR energy intake[tiab] OR Stressful event*[tiab] OR Life change*[tiab] OR Lifestyle*[tiab] OR Depression*[tiab] OR Depressive disorder*[tiab] OR Stress*[tiab] OR Anxiety[tiab] OR Postpartum[tiab] OR parenting[tiab]) *AND (publisher[sb] OR inprocess[sb])) AND ("1980/01/01"[PDat] : "3000/12/31"[PDat])*

5064 references from 1980 to 15.09.2016

5639 *references from 1980 to 24.01.2018 (Update)* *the terms in italics were added*

**Embase.com**

('pregnancy diabetes mellitus'/de AND ('physical activity, capacity and performance'/exp OR 'fitness'/de OR 'motor activity'/exp OR 'feeding behavior'/exp OR 'hunger'/de OR 'satiety'/de OR 'dietary intake'/exp OR 'diet'/exp OR 'body weight'/exp OR 'body composition'/exp OR 'lifestyle and related phenomena'/de OR 'life event'/de OR 'lifestyle'/de OR 'lifestyle modification'/de OR 'sedentary lifestyle'/de OR 'health behavior'/de OR 'attitude to health'/de OR 'health belief'/de OR 'depression'/exp OR 'stress'/exp OR 'child parent relation'/exp OR 'parenting education'/de OR 'anxiety'/de OR 'anxiety disorder'/exp OR 'puerperium'/exp OR 'meal'/de OR 'waist circumference'/de OR 'attitude to illness'/de)) NOT ([animals]/lim NOT [humans]/lim) *NOT 'conference abstract'/it) AND [1980-2018]/py*

9109 references from 1980 to 15.09.2016

7823 *references from 1980 to* *12.02.2018 (Update, conference abstracts not considered): the terms in italics were added*

**CINAHL**

MH "Diabetes Mellitus, Gestational" AND ((MH "Exercise+") OR (MH "Physical Activity") OR (MH "Physical Fitness+") OR (MH "Swimming") OR (MH "Cycling") OR (MH "Walking") OR (MH "Relaxation") OR (MH "Running+") OR (MH "Motor Activity") OR (MH "Eating Behavior+") OR (MH "Hunger") OR (MH "Appetite") OR (MH "Eating") OR (MH "Postprandial Period") OR (MH "Diet+") OR (MH "Food Intake+") OR (MH "Satiation") OR (MH "Dietary Fats+") OR (MH "Dietary Carbohydrates+") OR (MH "Meals+") OR (MH "Body Weight+") OR (MH "Waist Circumference") OR (MH "Waist-Hip Ratio") OR (MH "Body Composition+") OR (MH "Life Style+") OR (MH "Attitude to Health+") OR (MH "Attitude to Illness") OR (MH "Attitude to Obesity") OR (MH "Attitude to Pregnancy") OR (MH "Attitude to Change") OR (MH "Depression+") OR (MH "Anxiety") OR (MH "Stress+") OR (MH "Anxiety Disorders") OR (MH "Parenting") OR (MH "Parental Behavior") OR (MH "Paternal Behavior") OR (MH "Parent-Child Relations") OR (MH "Postnatal Period+"))

1204 references from 1980 to 15.09.2016

1400 references from 1980 to 25.01.2018 (Update)

**PsycINFO**

gestational diabetes/ AND (exp physical activity/ or active living/ or exp physical fitness/ OR sedentary behavior/ OR exp eating behavior/ or exp appetite/ or diets/ or eating attitudes/ or food intake/ OR satiation/ OR exp health attitudes/ or exp lifestyle/ or health behavior/ or exp major depression/ OR exp stress/ OR anxiety/ or exp anxiety disorders/)

43 references from 1980 to 15.09.2016

58 references from 1980 to 24.01.2018 (Update)

**Cochrane Library Wiley**

("Pregnancy-Induced" NEXT/1 Diabete* OR Gestational NEXT/1 diabete*) AND (Exercise OR Swimming OR Stretching OR Walking OR Physical NEXT/1 activit* OR Aerobic OR "Strength training" OR Sedentary OR inactivity OR Cycling OR running OR "resistance training" OR feeding NEXT/1 behav* OR eating NEXT/1 behav* OR Feeding NEXT/1 Pattern* OR Eating NEXT/1 Pattern* OR Food NEXT/1 habit* OR Eating NEXT/1 habit* OR Diet NEXT/1 habit* OR Dietary NEXT/1 habit* OR Breast NEXT/1 feeding OR Breastfeeding OR (Mindful* NEAR/3 eating) OR Intuitive NEXT/1 eating OR Food NEXT/1 intake OR Hunger OR satiation OR dietary NEXT/1 fat* OR diet OR diets OR sugar* OR carbohydrate* OR sucrose OR weight OR overweight OR obesity OR body NEXT/1 composition OR appetite OR energy NEXT/1 intake OR Stressful NEXT/1 event* OR Life NEXT/1 change* OR Lifestyle* OR Depression* OR Depressive NEXT/1 disorder* OR Stress* OR Anxiety OR Postpartum OR parenting):ab,ti

606 references from 1980 to 15.09.2016

Cochrane Database of Systematic Reviews : Issue 9 of 12, September 2016

Cochrane Central Register of Controlled Trials : Issue 8 of 12, August 2016

Database of Abstracts of Reviews of Effect : Issue 2 of 4, April 2015

824 references from 1980 to 24.01.2018 (Update)

Cochrane Database of Systematic Reviews : Issue 1 of 12, January 2018

Cochrane Central Register of Controlled Trials : Issue 12 of 12, December 2017
